# Mitochondrial Function and Protein Turnover in the Diaphragm are Altered in LLC Tumor Model of Cancer Cachexia

**DOI:** 10.3390/ijms21217841

**Published:** 2020-10-22

**Authors:** Megan E. Rosa-Caldwell, Conner A. Benson, David E. Lee, Jacob L. Brown, Tyrone A. Washington, Nicholas P. Greene, Michael P. Wiggs

**Affiliations:** 1 Exercise Science Research Center, Cachexia Research Laboratory, Department of Health, Human Performance and Recreation, University of Arkansas, Fayetteville, AR 72701, USA; merosaca@bidmc.harvard.edu (M.E.R.-C.); david.e.lee@duke.edu (D.E.L.); Jacob-brown@omrf.org (J.L.B.); npgreene@uark.edu (N.P.G.); 2 Integrative Physiology and Nutrition Laboratory Name, Department of Health and Kinesiology, University of Texas at Tyler, Tyler, TX 75799, USA; cbenson@patriots.uttyler.edu; 3 Exercise Science Research Center, Exercise Muscle Biology Laboratory, Department of Health, Human Performance and Recreation, University of Arkansas, Fayetteville, AR 72701, USA; tawashin@uark.edu; 4 Department of Health, Human Performance and Recreation, Baylor University, Waco, TX 76798, USA

**Keywords:** protein synthesis, protein degradation, muscle atrophy, mitochondrial function, mTOR, oxidative stress, FOXO

## Abstract

It is established that cancer cachexia causes limb muscle atrophy and is strongly associated with morbidity and mortality; less is known about how the development of cachexia impacts the diaphragm. The purpose of this study was to investigate cellular signaling mechanisms related to mitochondrial function, reactive oxygen species (ROS) production, and protein synthesis during the development of cancer cachexia. C57BL/J6 mice developed Lewis Lung Carcinoma for either 0 weeks (Control), 1 week, 2 weeks, 3 weeks, or 4 weeks. At designated time points, diaphragms were harvested and analyzed. Mitochondrial respiratory control ratio was ~50% lower in experimental groups, which was significant by 2 weeks of cancer development, with no difference in mitochondrial content markers COXIV or VDAC. Compared to the controls, ROS was 4-fold elevated in 2-week animals but then was not different at later time points. Only one antioxidant protein, GPX3, was altered by cancer development (~70% lower in experimental groups). Protein synthesis, measured by a fractional synthesis rate, appeared to become progressively lower with the cancer duration, but the mean difference was not significant. The development and progression of cancer cachexia induces marked alterations to mitochondrial function and ROS production in the diaphragm and may contribute to increased cachexia-associated morbidity and mortality.

## 1. Introduction

Although decades of cancer research have resulted in increased lifespan and longevity across a variety of cancers, cancer mortality remains one of the leading causes of death in Western nations [1,2]. One of the proposed reasons for this sustained cancer mortality is body weight and muscle loss, clinically diagnosed as cancer cachexia. Broadly defined, cancer cachexia is a co-pathology of cancer, characterized by systemic inflammation, increased protein degradation, and decreased protein synthesis [3,4,5]. These systemic alterations and subsequent muscle loss are associated with decreased quality of life and increased mortality in cancer patients, with at least 20% of cancer related deaths attributed to cachexia [4]. Importantly, cancer cachexia affects 20–89% of cancer patients depending on tumor type and progression [6,7,8,9]. These data demonstrate the significance of cachexia in the etiology of cancer development and the need for more research to understand mechanisms contributing to the development of cachexia.

While cachexia clearly impacts muscles required for general locomotion, given the systemic nature of cachexia [10], it is plausible cachexia also affects other non-locomotive muscles, such as the diaphragm. The diaphragm is critical for proper respiratory function. Importantly, intensive care unit (ICU) associated studies have clearly demonstrated diaphragm atrophy is a strong predictor of mortality in pre-clinical and clinical settings [11,12,13]. Indeed, recent works have suggested cancer cachexia to affect diaphragm size and function in murine models, with multiple studies finding ~25–30% diaphragm atrophy and corresponding force production in cachectic mice [14,15,16,17,18,19,20]. Additionally, human models appear to suggest pulmonary function and cachexia are closely related [21]. However, mechanisms contributing to atrophy in locomotor-specific skeletal muscle and the diaphragm may differ. For example, some works have found alterations to reactive oxygen species neutralizing capacity and protein oxidation in the skeletal muscle but not in the diaphragm in cachectic mice [14,19]. More so, other works have not found aberrations to diaphragm size or force production in cachexia, though it should be noted these were mildly cachectic animals [22,23]. Taken together, these data suggest diaphragm alterations during cachexia development are nuanced and may be different as the diseases progresses.

The time-course of alterations occurring in the diaphragm during cachexia development may be an important consideration for defining the etiology of diaphragm atrophy and important in preventing or treating cachexia. For example, literature over the past decade has demonstrated that energy metabolism, mitochondrial bioenergetics, and inflammatory signaling are altered before the onset of cachexia, with some cellular aberrations occurring before palpable tumors develop [10,24]. Moreover, recent works have speculated that once cachexia develops, it may be irreversible [25,26,27]. Therefore, it may be more prudent to seek therapeutics that can prevent the development of cachexia. In order to develop these effective therapeutics, more research understanding the development and progression of muscle atrophies is imperative. Therefore, the purpose of this study was to investigate cellular signaling cascades related to muscle atrophy in the diaphragm throughout the development and progression of Lewis lung carcinoma-induced cancer cachexia in mice.

## 2. Results

Data from these animals demonstrating the hallmark of cachexia, limb muscle wasting, and lower fat mass, have been previously published [24,28], with 4 week animals having 15% lower tibialis anterior (TA) mass, 12% lower plantaris masses, 13% lower gastrocnemius, and 35% lower fat mass compared to 0 week animals. It is important to note that muscle mass in 1, 2, and 3 weeks were not significantly different from 0 week controls.

### 2.1. Lewis Lung Carcinoma (LLC) Resulted in Alterations to Mitochondrial Function without Changes to Mitochondrial Content

**Figure 1:**
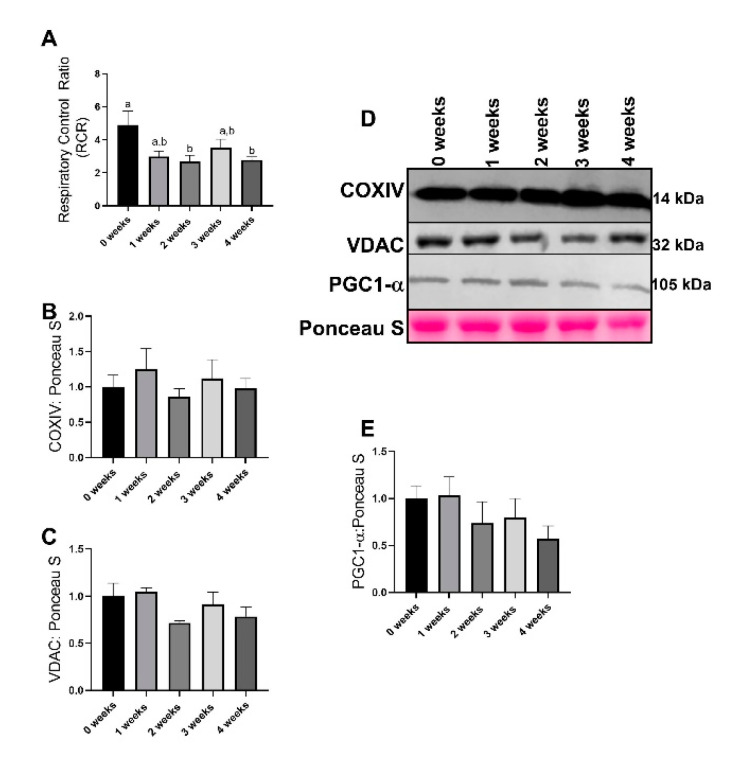
Mitochondrial function as related to respiratory control ratio (RCR) and markers of mitochondrial content and biogenesis. (**A**) Mitochondrial state 3: state 4 respiration, also known as respiratory control ratio (RCR). (**B**) COXIV (Mitochondrial cytochrome c oxidase subunit IV) protein content. (**C**) VDAC (Voltage-dependent anion channel) protein content. (**D**) Representative images for immunoblotting from the current study. (**E**) PGC1α protein content. Data are presented as mean ± SEM. Different letters represent statistical differences at a Tukey adjusted *p* < 0.05. n = 8–10/group.

**Figure 2:**
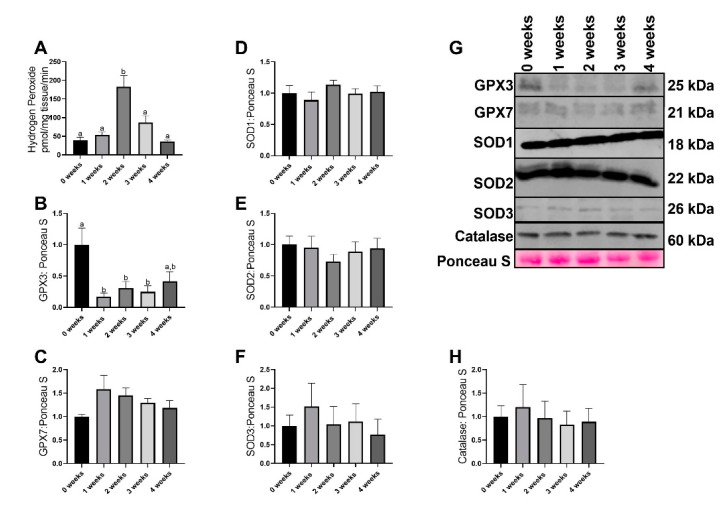
Reactive oxygen species generation and immunoblot data of antioxidant proteins (**A**) Hydrogen peroxide content (reactive oxygen species). (**B**) GPX3 protein content. (**C**) GPX7 protein content. (**D**) SOD1 protein content. (**E**) SOD2 protein content. (**F**) SOD3 protein content. (**G**) Representative images of immunoblotting data. (**H**) Catalase protein content. Data are presented as mean ± SEM. Different letters represent statistical differences at a Tukey adjusted *p* < 0.05. n = 8–10/group.

Mitochondrial respiratory control ratio (RCR) was used as a measure of mitochondrial function. This method has previously been noted as the most accurate marker for mitochondrial function [29]. RCR values demonstrated statistical significance (*p* = 0.016), with 2 week and 4 week group animals having ~50% lower RCR values compared to 0 week (Figure 1A). Additionally, both 1 week and 3 week animals had similar patterns to RCR, though the pairwise differences did not reach statistical significance (*p* = 0.067 and *p* = 0.324 respectively, Figure 1A). There were no differences between groups in mitochondrial content measured by either COXIV (Mitochondrial cytochrome c oxidase subunit IV) (*p* = 0.765, Figure 2B,D) and VDAC (Voltage-dependent anion channel) (*p* = 0.144, Figure 2C,D). There were no differences between groups in protein content of PGC1α, a surrogate marker of mitochondrial biogenesis (*p* = 0.381, Figure 2D,E).

### 2.2. LLC Resulted in Greater Reactive Oxgyen Species Generation without any Changes in most Antioxidant Related Proteins

Mitochondrial reactive oxygen species production was altered with the progression of cachexia (*p* < 0.0001), with 2 week animals having ~2.5-fold greater reactive oxygen species (ROS) production compared to all other experimental groups (Figure 2A). GPX3 demonstrated significant (*p* = 0.005) alterations with 1 week, 2 week, and 3 week animals having ~70% lower GPX3 protein content compared to 0 week (Figure 2B,G). Additionally, the difference between 0 week and 4 week animals approached statistical significance (*p* = 0.079, Figure 2B,G). GPX7 protein content did not differ between groups (*p* = 0.168, Figure 2C,G). There were no differences in SOD1 protein content across any groups (*p* = 0.584, Figure 2D,G). No differences were noted in SOD2 protein content across any groups (*p* = 0.782, Figure 2E,G). There were no group differences in SOD3 protein content (*p* = 0.832, Figure 2F,G). No differences were noted in Catalase protein content across all groups (*p* = 0.953, Figure 2H,G).

### 2.3. LLC Did not Have Significant Effects on Protein Synthesis Signaling

**Figure 3:**
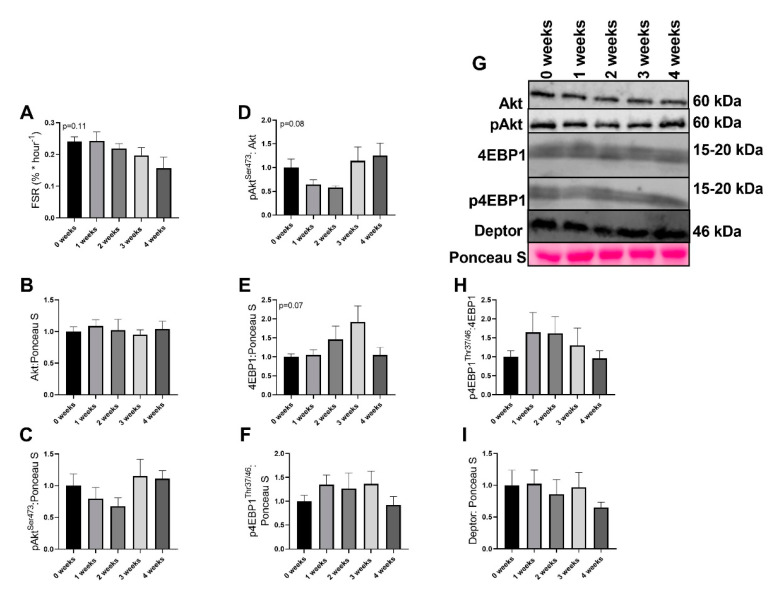
Markers of protein anabolism in the diaphragm. (**A**) Fractional synthesis rate (FSR) (**B**) Akt protein content (**C**) pAkt^Ser473^ protein content. (**D**) pAkt^Ser473^/Akt protein content. (**E**) 4EBP1 protein content (**F**) p4EBP1^Thr37/46^ protein content. (**G**) Representative images for immunoblot data (**H**) p4EBP1^Thr37/46^/4EBP1 protein content. (**I**) Deptor protein content. Data are presented as mean ± SEM. n = 8–10/group.

Although the global F test was not significant (*p* = 0.11), there appeared to be a progressive loss of mixed muscle fractional synthesis rate (FSR) with the development of cachexia (Figure 3A). There were no differences noted in Akt or pAkt^Ser473^, (*p* = 0.947 and *p* = 0.328, respectively, Figure 3B,C,G). However, pAkt^Ser473^/Akt ratio approached statistical significance (*p* = 0.0850, Figure 3D,G), yet no pairwise differences were noted. 4EBP1 approached statistical significance (*p* = 0.074, Figure 3E,G); however, no pairwise differences were noted. p4EBP1^Thr37/46^ was not different between any groups (*p* = 0.523, Figure 3F,G). p4EBP1/4EBP1^Thr37/46^ ratio did not differ between groups (*p* = 0.567, Figure 3H,G). There were no differences in Deptor content across any groups (*p* = 0.726, Figure 3I,H).

### 2.4. LLC Appeared to Increase FOXO3-Dependent Signaling

**Figure 4:**
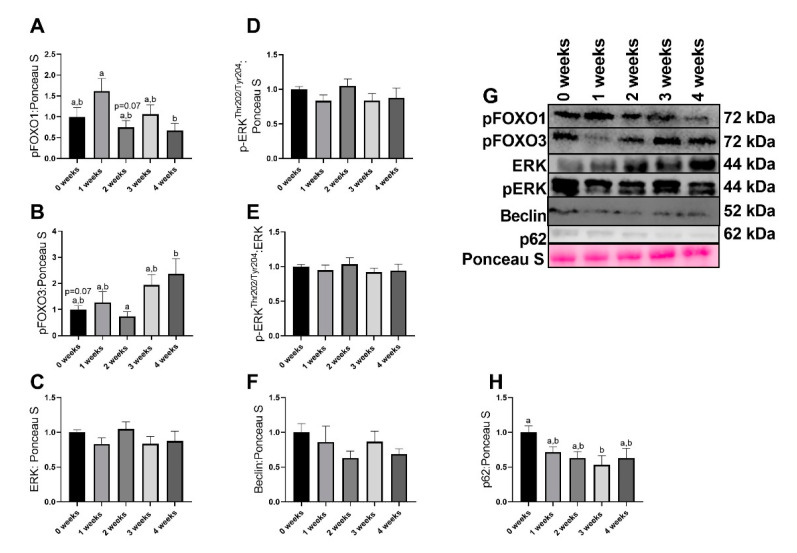
Immunoblot data for moderators of protein anabolism. (**A**) pFOXO1 protein data. (**B**) pFOXO3 protein content. (**C**) ERK protein content. (**D**) pERK^Thr202/Tyr204^. (**E**) pERK^Thr202/Tyr204^/ERK protein content. (**F**) Beclin protein content. (**G**) Representative images for immunoblot data. (**H**) p62 protein data. Data are presented as mean ± SEM. Different letters represent statistical differences at a Tukey adjusted *p* < 0.05. n = 8–10/group.

Significant differences were noted in pFOXO1 content (*p* = 0.040), with 1 week animals having ~2-fold greater pFOXO1 protein content compared to 4week (*p* = 0.034). Additionally, the difference between 1 week and 2 week animals (~80% greater pFOXO1 in 1 week compared to 2 week) approached statistical significance (Figure 4A,G). Significant differences were also found in pFOXO3 content (*p* = 0.023), with 4 week animals having ~1.3-fold greater pFOXO3 content that approached statistical significance (*p* = 0.071, Figure 4B,G). There were no alterations in total ERK protein content (*p* = 0.441, Figure 4C,G). There were no differences in pERK^Thr202/Tyr204^ protein content across any groups (*p* = 0.441, Figure 4D,G). There were no differences in pERK/ERK ratio across any groups (*p* = 0.822, Figure 4E,G). There were no differences in Beclin protein content across any groups (*p* = 0.400, Figure 4F,G). In p62 protein content, the global F test approached statistical significance (*p* = 0.055), with 3 week animals having ~45% lower p62 protein content compared to 0 week animals (Figure 4H,G).

## 3. Discussion

Cancer cachexia is an important clinical problem that lacks a therapeutic intervention capable of preventing or treating the condition. Cachexia research in the diaphragm traditionally focuses on the endpoint of the condition, without describing the metabolic changes occurring in the muscle; therefore, we conducted a four-week time course study to identify the changes that occur leading up to a cachectic phenotype. We report that mitochondrial alterations, specifically mitochondrial respiration, is severely limited early in the development and progression of cachexia within the diaphragm. These alterations occur before any other cellular signaling cascades that may lead to muscle atrophy. Taken together this study further highlights the role of mitochondrial dysfunction as a possible initiating factor in the etiology of protein catabolism and subsequent muscle loss during cancer cachexia.

One of our most robust findings is the substantial reduction in mitochondria function measured by RCR. Interestingly, these aberrations occurred extremely early in the development of cachexia, well before notable muscle wasting in these same animals [24,28]. In fact, compared to our previous works, significant reductions in mitochondrial efficiency occurred in the diaphragm before the limb muscle [24], perhaps suggesting that the diaphragm may be more susceptible to mitochondrial aberrations during cachexia development. This would align with research across other models of myopathies, such as in critical care patients [13,18,30] as well as works demonstrating differential responses between tissues during cachexia and other inflammatory diseases [17,31,32,33]. This divergent response may be due to different fiber type compositions, as the prior works were conducted in more mixed muscle fibers (plantaris and gastrocnemius), compared to the highly oxidative diaphragm muscle. Regardless, the diaphragm and appendicular skeletal muscle appear to have differential responses to atrophic conditions and warrants further investigation. It is plausible that a decrease RCR is accompanied by reduced ATP production despite no changes in mitochondrial content (COXIV, VDAC, and PGC1-α), implying a reduction in overall mitochondrial efficiency. Other works in inflammatory models have found reductions in mitochondrial ATP production without changes in mitochondrial content in the diaphragm [33], suggesting an overall discoordination of ATP production and mitochondrial content. Importantly, these alterations to mitochondrial respiration coincide with reductions in diaphragm function [17,33,34]. Although we did not directly measure diaphragm function or size, taken together our results appear to suggest synchronization between mitochondrial efficiency and diaphragm functionality during inflammatory pathologies [21,35].

In addition to reductions in mitochondrial function, we find substantial ROS generation in the diaphragm during early phases of cancer development. ROS generation was greater at two weeks compared to other groups. This finding aligns with prior works in these same animals, with greater ROS production 1–2 weeks after tumor implantation in the limb muscle. Together these results suggest that ROS production may be causative for the development of protein catabolism and not necessarily a consequence of catabolism. More so, we and others [24,33] do not find any concurrent changes in proteins responsible for ROS neutralization, with the notable exception of GPX3 in the current investigation. Recent works have determined the strong regulatory function of GPX3 in mitochondrial morphology, ROS production, and mitochondrial polarization [36]. Specifically, loss of GPX3 is associated with increased ROS generation in yeast cells and subsequent aberrations to mitochondrial morphology and function [36]. This work, in conjunction with our data, may suggest cachexia induces loss of GPX3 content, which in turn may facilitate ROS generation and subsequent pathologies. This aligns some of the time course of alterations, with GPX3 content falling within one week of tumor development and ROS generation occurring at two weeks. However, we were not able to delineate mitochondrial vs. cytosolic GPX3 which could influence the interpretation of the results, as mitochondrial GPX3 appears to be the more important component for mitochondrial morphology [36]. Additionally, based on this interpretation, we would expect elevated ROS throughout the duration of cachexia, which we did not see, so more work is necessary to understand the role of ROS generation within the diaphragm during cachexia development. Regardless, the greater ROS production without any changes in antioxidant proteins suggests that during cachexia development muscle does not appear to respond to increased oxidative stress with upregulation of antioxidant content, which may contribute to eventual protein catabolism and muscle atrophy. However, we should acknowledge protein content does not necessarily indicate activity.

While we find noted alterations in mitochondrial efficiency and ROS generation, we do not find robust alterations to markers of protein turnover. The alterations in the fractional synthesis rate did not reach statistical significance yet suggested an eventual trend for reduced protein synthesis. In addition, we find greater content in markers that suggest a potential increase in catabolic signaling cascades. For example, we find lower content in autophagy marker p62. p62 is a cargo protein responsible for shuttling proteins to the autophagosome for autophagy-mediated degradation [37]. Therefore, reduced p62 protein content is often interpreted as increased autophagy resolution. The lower p62 protein content we found in experimental groups may suggest upregulation of degradative pathways in the diaphragm of cachectic animals. Indeed, prior works have found greater degradative pathways in the diaphragm during cachexia [18,38,39]. However, we must acknowledge that overall we did not find robust alterations to regulators of protein turnover. Interestingly, recent works have demonstrated that in the PDX model of cancer cachexia, genes related to protein turnover are not as strongly altered compared to skeletal muscle [32]. These results once again suggest the diaphragm may have a separate etiology for cachexia development compared to skeletal muscle and warrants further research for other tissues during the development and progression of cancer cachexia.

A notable limitation of the present study is that we were unable to directly measure diaphragm and respiratory function during this study. However, many prior works have noted alterations to diaphragm function in murine models of cancer cachexia including lower fiber cross sectional area, deteriorations in contractile efficiency, and altered ventilatory responses [18,40]. Taken together, although we did not specifically measure ventilatory function in the present study, it is plausible to infer that these same detriments to diaphragm health occurred with the onset of cachexia (~3–4 weeks [24,28]).

In conclusion, this work provides novel data demonstrating early mitochondrial alterations in the diaphragm during cachexia escalation. The initiation of mitochondrial aberrations, in turn, potentially results in diaphragm atrophy. Consequently, this diaphragm atrophy plausibly leads to breathing difficulties and increased morbidity for cancer patients. Importantly, these data suggest systemic aberrations occur quite early during cancer growth and highlight the necessity for therapeutic interventions early on during cancer development.

## 4. Materials and Methods

### 4.1. Animals

We have previously reported on aspects of limb, hepatic, and cardiac muscles from these animals [24,28,41,42]. All protocols and procedures were approved by the University of Arkansas Institutional Animal Care and Use Committee (Protocol number: 15065, July 2015). Male C57BL/6J mice were purchased from Jackson Laboratories (stock 000664). Animals were kept in a temperature and humidity-controlled facilities at the University of Arkansas and maintained under a 12 h light: 12 h dark cycle. Animals were fed standard laboratory chow diet (17% fat, 54% CHO, and 29% protein, 70% carbohydrate, 3.0 kcal/gram, Teklad, Indianapolis, IN, Product #8640). Throughout the duration of the study, animals were checked daily for general health and signs of distress. If any animal demonstrated excessive tumor burden (inability to ambulate) or tumor necrosis they were euthanized and removed from the study.

### 4.2. Study Design

At eight weeks of age, animals were implanted with either phosphate buffered saline (PBS) or 1 × 10^6^ Lewis Lung Carcinoma (LLC) cells (ATCC, Manassas, VA) in the left hind flank. LLC was allowed to develop for 1, 2, 3, or 4 weeks [24,28]. PBS animals were age matched to 4-week animals, creating five experimental groups: PBS, 1 week, 2 week, 3 week, and 4 week (n = 12–24/group) [24,28]. This design allowed us to investigate how different outcomes measures changed with prolonged duration of cancer development. At the end of designated time points, animals were anesthetized with 3% isoflurane. Complete anesthesia was confirmed by lack of response to toe pinch. Diaphragms were collected and a portion of the diaphragm was snap frozen in liquid nitrogen and stored in −80 degree freeze for subsequent analysis. Another portion of the diaphragm was analyzed for mitochondrial and ROS production (n = 8–12/group, described below). After collection of tissues, while still under anesthesia, animals were euthanized through aortic puncture. A subset of 8–10 animals/group were selected for subsequent Western blot analysis of cellular function and signaling.

### 4.3. Determination of Muscle Protein Synthesis

Fractional protein synthesis rates (FSR) within the skeletal muscle was determined using deuterium oxide (^2^H_2_O) precursor-product labeling as previously described [28,43]. 24 h prior to tissue harvest, mice were injected with 99.9% ^2^H_2_O in the peritoneum (20 μL/g body weight, Sigma-Aldrich, St. Louis, MO, USA). Cage drinking water was supplemented with 4% ^2^H_2_O in H_2_O to maintain the precursor pool. Following the 24-h enrichment, animals were anesthetized, blood drawn from cardiac puncture, centrifuged, and plasma was frozen at −80 °C.

Muscle enrichment for mixed protein synthesis was completed using ~10 mg of diaphragm. Briefly, diaphragms were homogenized in a 10% trichloroacetic acid (TCA) solution, afterwards samples were centrifuged, and soluble cytosolic amino acids were decanted. Samples were then incubated in 6 M hydrochloric acid (HCl) at 100 °C to hydrolyze proteins into amino acids. A portion of the hydrolysate was dried down and derivatized with a 3:2:1 v/v solution of methyl-8, methanol, and acetonitrile to determine ^2^H-labelling of alanine. 1 μL of solution was analyzed in a GC–MS capillary column (Agilent 7890A GCMS with a HP-5 ms capillary column) at an 80:1 split. Full GC–MS settings have previously been described [28,43].

Free ^2^H_2_O within the plasma was converted to acetone using 5% v/v solution of acetone/acetonitrile and 10 M NaOH for 24 h. The reaction was stopped by adding ~0.5g Na_2_SO_4_ and 0.6 mL chloroform. The solution was then analyzed using the GC–MS at a 20:1 split.

FSR of mixed proteins was calculated using the equation EA × [EBW × 3.7 × t (h)] − 1 × 100. Specifically, EA represented the protein bound 2H alanine, EBW was the total ^2^H_2_O in body water, 3.7 represented the exchange of ^2^H between body water and alanine, and t represented the time the ^2^H was present in the body in hours.

### 4.4. Mitochondrial Function

Mitochondrial function was measured as previously described [24,44]. Approximately 25 mg of costal diaphragm muscle was dissected and placed on a plastic Petri dish containing ice-cold Buffer X (60 mM K-MES, 35 mM KCl, 7.23 mM K_2_EGTA, 2.77 mM CaK_2_EGTA, 20 mM imidazole, 0.5 mM DTT, 20 mM taurine, 5.7 mM ATP, 15 mM PCr, and 6.56 mM MgCl2, pH 7.1). The muscle was trimmed of connective tissue and cut down to fiber bundles (4–8 mg wet weight). The muscle fiber bundles were gently separated in ice-cold buffer X to maximize surface area of the fiber bundle. To permeabilize the myofibers, each fiber bundle was incubated in ice-cold buffer X containing 50 μg/mL saponin on a rotator for 30 min at 4 °C. The permeabilized muscle bundles were then washed in ice-cold Buffer Z (110 mM K-MES, 35 mM KCl, 1 mM EGTA, 5 mM K2HPO4, and 3 mM MgCl_2_, 0.005 mM glutamate, and 0.02 mM malate and 0.5 mg/mL BSA, pH 7.1).

Mitochondrial respiration was measured using respiration chambers (Hansatech Instruments, King’s Lynn, Norfolk, England) maintained at 37 °C. Following calibration of the Clark oxygen electrode, permeabilized fiber bundles were incubated with 1 mL of respiration buffer containing 20 mM creatine to saturate creatine kinases. Flux through complex was measured by adding using 5 mM pyruvate and 2 mM malate. The ADP-stimulated respiration (state 3) was initiated by adding 0.25 mM ADP to the respiration chamber. Leak respiration (state 4) was determined in the presence of 10 μg/mL oligomycin to inhibit ATP synthase. The respiratory control ratio (RCR) was calculated as the ratio of state 3/state 4 respiration with a greater RCR indicative of greater oxygen consumption and ATP synthesis.

### 4.5. Reactive Oxygen Species (ROS) Emission

Mitochondrial ROS emission from a permeabilized fiber bundle was determined using the Amplex Red reagent (Life Technologies, Carlsbad, Californa) (7, 22, 23). Permeabilized fibers were incubated in Buffer Z along with Amplex Ultra Red (AR) (Thermo Fisher Scientific, Waltham, MA, Cat# A36006). Hydrogen peroxide reacts with AR and horseradish peroxidase (HRP) to produce resorufin (excitation wavelength 563 nm, emission 587 nm), in a 1:1 ratio. Fluorescence was quantified using Maya LSL Spectrometer (Ocean Optics, Dunedin Florida) with the appropriate excitation source. Data was collected Ocean View (Ocean Optics, Dunedin, Florida) and analyzed using Pasco Capstone Software (Roseville, California). Baseline fluorescence (respiration buffer, permeabilized fibers, HRP, and AR) for 8 min and then H_2_O_2_ production was increased by the addition of succinate (final concentration of 10 mM). The slopes at baseline and succinate-stimulated were measured, converted to H_2_O_2_ concentrations based on H_2_O_2_-derived standard curve and corrected by dry tissue mass.

### 4.6. Western Blotting

Immunoblotting of proteins related to muscle protein synthesis and degradation were completed as previously described [45,46,47]. Protein concentrations were determined using an RC/DC commercial kit (Bio Rad, Hercules, CA, Cat# 5000121) and standard software (Gen5, BioTek, Winooski, VT). 40 µg of protein sample was loaded into SDS/Polyacrylamide gels and transferred onto polyvinylidene difluoride (PDVF) membranes. Membranes were then probed for proteins related to protein synthesis and degradation. Antibodies included: COXIV (Cell Signaling, Cat# 4844S), VDAC (Cell Signaling, Cat# 4866S), PGC1α (Santa Cruz, Cat# sc-13067), GPX3 (GeneTex, Cat# GTX89142), GPX7 (GeneTex, Cat# GTX117516), SOD1 (Genetex, Cat# GTX100554), SOD2 (Cell Signaling, Cat# 131945), SOD3 (R & D Systems, Cat# AF4817), Catalase (Cell Signaling, Cat# 140975), Akt (Cell Signaling, Cat# 9272), pAkt^Ser473^ (Cell Signaling, Cat# 9271), 4EBP1 (Cell Signaling, Cat# 9452), p4EBP1^Th37/46^ (Cell Signaling, Cat# 9451), pFOXO1^T24^ (Cell Signaling, Cat#9464), pFOXO3^T32^ (Cell Signaling, Cat# 9464), ERK (Cell Signaling, Cat# 4695), pERK^Thr202/Tyr204^ (Cell Signaling, Cat# 4370), p62 (Sigma, Cat# p0067), Beclin (Cell Signaling, Cat# 3738), and Deptor (EMD Millipore, Cat# ABS222). Membranes were then imaged using a Li-Cor Odyssey imaging system and analyzed using Image Studio Lite Software (Li-Cor, Lincoln, NE, USA).

### 4.7. Statistical Analysis

All data were analyzed by one-way ANOVA using the PROC MIXED function in SAS (SAS Institute, Cary, North Carolina) for with independent factors cancer progression time (0 weeks vs. 1 week vs. 2 weeks vs. 3 weeks. vs. 4 weeks). When significant F ratios were noted, pairwise differences were analyzed using a Tukey post-hoc analysis. Significance was denoted as *p* ≤ 0.05.
